# Non-renewal spiking and neural dynamics - a simple theory of interspike-interval correlations in adapting neurons

**DOI:** 10.1186/1471-2202-14-S1-O9

**Published:** 2013-07-08

**Authors:** Tilo Schwalger, Benjamin Lindner

**Affiliations:** 1 Department of Physics, Humboldt-Universität zu Berlin, Berlin, 12489, Germany; 2 Bernstein Center for Computational Neuroscience, Berlin, 10115, Germany

## 

**Figure 1:**
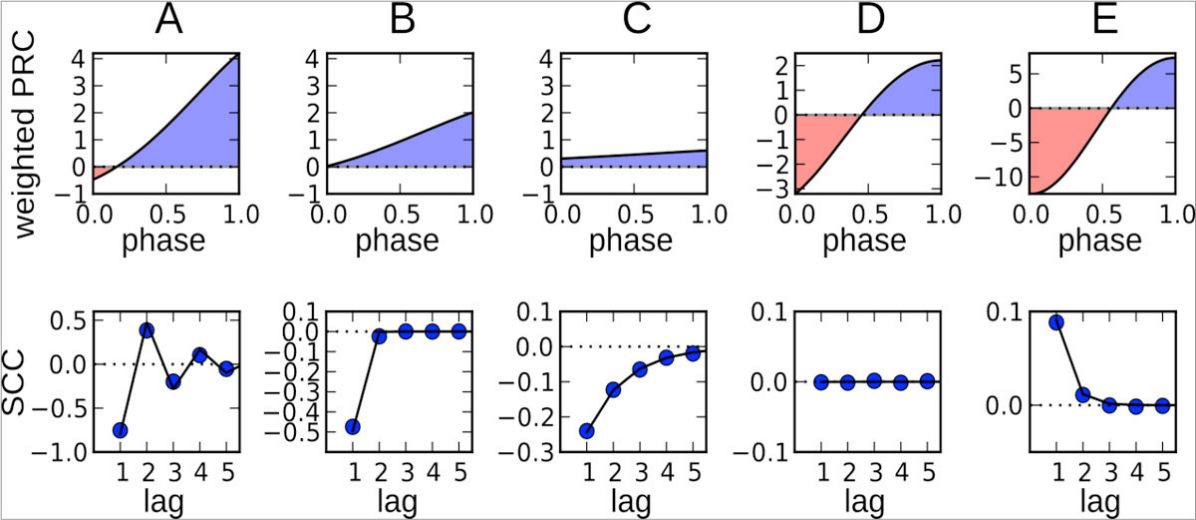
**Possible patterns of ISI correlations of an adapting neuron (bottom)**. The correlation pattern is determined by the area under the weighted PRCs (top). Shown are data for a two-dimensional noisy resonate-and-fire model with adaptation (simulations: circles, theory: solid line).

There is accumulating evidence that the spiking of many neurons is not a renewal process but is characterized by correlations between interspike intervals (ISIs) [1]. These correlations are crucial for understanding signal processing in single neurons, however, their origin and structure is still poorly understood theoretically. Here, we present a simple theory of correlations in neural oscillators with spike-triggered adaptation currents, which are a major source of non-renewal spiking. These currents mediate spike-frequency adaptation and are commonly believed to result in negative correlations between adjacent ISIs. For such adapting neurons, we show that the serial correlation coefficient (SCC) is fundamentally related to the neuron's phase response curve (PRC). The relation predicts possible correlation patterns that characterize how correlations depend on the lag between ISIs. Different patterns arise from the specific interplay between nonlinear neural dynamics and adaptation dynamics. In particular, the correlation structure can be determined by a single parameter that includes the shape of the PRC as well as the strength and time scale of the adaptation current (Fig.1).

For a positive PRC (type I), the SCC is always negative at lag 1. At higher lags, we find either a monotonically decaying (Fig.1A) or oscillating (Fig.1C) behavior of the SCC depending on the strength of adaptation. Similar correlation structures have been observed in different experimental studies (see e.g. [2]). Despite the distinct patterns, the total correlation as expressed by the sum of the SCC over all lags displays a universal value close to -0.5 at high firing rates and strong adaptation. As an example of a type I neuron, we discuss one-dimensional integrate-and-fire (IF) neurons with adaptation (like e.g. the adaptive exponential IF model [3]) operating in the supra-threshold regime. For these models, the different behaviors of the SCC can be explained by qualitatively different structures of the phase plane spanned by the voltage and the adaptation variables. Our theory also predicts that adapting neurons with a partly negative PRC (type II phase resetting) can additionally exhibit non-negative ISI correlations, which is indeed found in a two-dimensional IF model with adaptation (Fig.1D,E). Thus, adapting neurons can show a richer repertoire of correlation patterns than previously thought.
